# Cross-cultural validation of the German and Turkish versions of the PHQ-9: an IRT approach

**DOI:** 10.1186/s40359-018-0238-z

**Published:** 2018-06-05

**Authors:** Hanna Reich, Winfried Rief, Elmar Brähler, Ricarda Mewes

**Affiliations:** 1 Department of Psychology, University of Marburg, Marburg, Germany; 2 Outpatient Unit for Research, Teaching and Practice, Faculty of Psychology, University of Vienna, Renngasse 6-8, 1010 Vienna, Austria; 3 Institute of Medical Psychology, Medical School, University of Leipzig, Leipzig, Germany; 4 Clinic and Policlinic for Psychosomatic Medicine and Psychotherapy, University Medical Center Mainz, Mainz, Germany; 5 Institute of Medical Psychology, Justus-Liebig-University, Gießen, Germany

**Keywords:** Depression, Patient health Questionnaire-9 (PHQ-9), Item response theory (IRT), Differential item functioning (DIF), Cross-cultural / ethnic comparison

## Abstract

**Background:**

The Patient Health Questionnaire’s depression module (PHQ-9) is a widely used screening tool to assess depressive disorders. However, cross-linguistic and cross-cultural validation of the PHQ-9 is mostly lacking. This study investigates whether scores on the German and Turkish versions of the PHQ-9 are comparable.

**Methods:**

Data from Germans without a migration background (German version, *n* = 1670) and Turkish immigrants in Germany (either German or Turkish version, *n* = 307) were used. Differential Item Functioning (DIF) was assessed using Item Response Theory (IRT) models.

**Results:**

Several items of the PHQ-9 were found to exhibit DIF related to language or ethnicity, e.g. ‘sleep problems’, ‘appetite changes’ and ‘anhedonia’. However, PHQ-9 sum scores were found to be unbiased, i.e., DIF had no notable impact on scale levels.

**Conclusions:**

PHQ-9 sum scores can be compared between Turkish immigrants and Germans without a migration background without any adjustments, regardless of whether they complete the German or the Turkish version.

## Background

Depression is a highly prevalent disorder leading to suffering and disability [1, 2]. It is predicted to be the major cause of burden of disease by 2020 [3]. Differences exist across countries and ethnic groups in epidemiology [4–7] and symptom presentation [8–10] of depressive disorders. Many cross-cultural studies applied self-report questionnaires to assess and describe the phenomenology of depressive disorders. However, cross-linguistic and cross-cultural validation of self-report questionnaires is mostly lacking. Such validation analyses are urgently needed for a valid comparison of prevalence rates and symptom profiles of depressive disorders across linguistic and ethnic groups [11]. Among self-report questionnaires for assessing depression, the Patient Health Questionnaire-9 (PHQ-9) [12, 13] is one of the most frequently used and best validated questionnaires worldwide [14–16]. It is recommended as a general measure of depression severity by the DSM-5 (Diagnostic and Statistical Manual of Mental Disorders, 5th Edition) [17] and has been translated into over 70 languages and dialects [18]. In the present study, we investigate whether PHQ-9 scores are comparable between the German majority population without a migration background and the largest minority group in Germany, Turkish immigrants [19].

To our knowledge, only three studies have investigated the comparability of different language versions of the PHQ-9: Huang and colleagues [20] found differences in item functioning between the English and Chinese version of the items assessing sleep, appetite, and psychomotor changes in a large sample of primary care patients. Comparing the English and Spanish version, they also found differences in sleep and appetite items, plus anhedonia and self-esteem items. Arthurs and colleagues [21] found differences between the English and French version for anhedonia, sleep, and self-esteem items in patients with systemic sclerosis. Comparing the German and Russian version in primary care patients [22], a difference in item functioning was found in the sleep problems item.

Regarding the comparability across ethnic and racial groups, two studies have confirmed the comparability of the English version between African-American and non-Hispanic White primary care patients [20, 23]. Moreover, one study in a general population sample confirmed the comparability of the German version between Germans without a migration background and a heterogeneous sample of immigrants living in Germany [24]. However, Crane and colleagues found differences in items about sleep, low energy, and psychomotor changes between HIV-infected African-Americans and Whites in the English version [25], and Baas and colleagues confirmed a cultural bias in the Dutch version of the PHQ-9 in the item psychomotor changes between Surinam Dutch and Native Dutch male primary care patients [11]. Although the reasons for differences in item functioning are mostly unclear, most studies confirmed that such differences had minimal impact on the scale level and that sum scores were mainly comparable across the investigated samples.

To establish cross-linguistic and cross-cultural measurement equivalence, equality in item functioning needs to be inspected. The probability of endorsing a specific item should be the same for all individuals with a certain underlying level of depression, and should not be influenced by ethnic or linguistic group. If these prerequisites are not fulfilled, the item is considered to have Differential Item Functioning (DIF) [26, 27]. The absence of DIF justifies cross-cultural comparisons based on the sum score as an indicator for the latent trait, and allows observed differences to be related to actual differences between groups. DIF can be appropriately assessed using Item Response Theory (IRT) analysis [28, 29]. IRT provides parametric and nonparametric models, which constitute powerful tools for separating measurement bias from true group differences [30, 31].

The objective of this study is to investigate whether PHQ-9 scores are comparable between Turkish immigrants in Germany and Germans without a migration background. This is especially important since Turkish immigrants represent the largest minority group in Germany [19], and are among the three largest immigrant populations in other European countries such as the Netherlands, Denmark, and Austria [32]. Moreover, as prevalence rates of affective disorders in labor migrants in Europe are elevated [5, 33, 34], properly working assessment instruments for depression are particularly important in this group.

First, we examine whether the German and Turkish language versions of the PHQ-9 are comparable. Then, we examine whether the German PHQ-9 is comparable across ethnic groups. This two-step approach is necessary because Turkish language utilization and German language proficiency vary considerably among Turkish immigrants [35]. Based on previous studies on DIF in PHQ-9 items, one might expect DIF in the sleep, psychomotor changes, anhedonia, appetite changes, and low self-esteem items. However, this is the first study to investigate cross-linguistic and cross-cultural validity of the Turkish version of the PHQ-9, and one of the few to study this topic at all. Consequently, all items of the PHQ-9 were tested on DIF without statistical pre-assumptions. Based on the results, recommendations for applying the PHQ-9 in Turkish immigrants are provided.

## Methods

### Data sources

This article provides secondary analyses of original data obtained in four independent, cross-sectional studies.

#### Study 1

A representative sample of the German general population (*n* = 2510) was screened for disability, somatic complaints, mental health, and healthcare utilization. The assessment was conducted by a demographic consulting company (USUMA, Berlin) in 2007. The study material was available in German only. Details of the procedure are described elsewhere, e.g. [36]. For the present analyses, only data of Germans without a migration background and of Turkish immigrants responding to the German language version of the PHQ-9 are used.

#### Study 2

A convenience sample of Turkish immigrants (*n* = 214) completed questionnaires about perceived discrimination and depressive and somatoform symptoms. Data were collected in 2011 and 2012 [37]. The study material was provided in German or Turkish according to the participants’ choice. The study was carried out using an online survey and paper-and-pencil versions with a snowball system.

#### Study 3

Two matched inpatient samples (Turkish immigrants vs. Germans without a migration background, *n* = 50 each) were recruited in five psychiatric clinics in 2011 and 2012 [38]. Participants were asked about subjective concepts of mental illness, motivation for psychotherapy, and mental health symptoms. The study material was provided as paper-and-pencil versions in German or Turkish according to the participants’ choice. A bilingual research assistant helped illiterate participants.

#### Study 4

In a pilot study, Turkish immigrant inpatients (*n* = 29) were recruited to participate in a randomized controlled trial (RCT) on the effects of a motivation-enhancing program at the beginning of their inpatient treatment. They provided baseline information about motivation for psychotherapy, mental health symptoms, and illness perception at the beginning of inpatient treatment in two different psychiatric clinics in 2013 and 2014. Study material was available on a computer in German or Turkish according to the participants’ choice. A bilingual research assistant helped participants who were illiterate or needed assistance with the computer. This sample was included to enclose Turkish immigrants with a low level of literacy in the analysis. Persons with low German language proficiency and low educational levels usually get excluded from research in Germany, but are characteristic for the population of Turkish immigrants [39].

### Measures

Participants in all studies provided information on sociodemographic and migration-related variables, and symptoms of depression measured by the PHQ-9. The PHQ-9 is a nine-item self-rating instrument, with each item representing one of the DSM-IV (Diagnostic and Statistical Manual of Mental Disorders, 4th Edition) criteria for a depressive episode (anhedonia, depressed mood, sleep problems, feeling tired, change in appetite, negative self-evaluation, concentration problems, psychomotor changes, suicidality). Each item can be scored as 0 (not at all), 1 (several days), 2 (more than half the days), or 3 (nearly every day), according to the frequency of experiencing difficulties in the respective area in the previous 2 weeks. Sum scores range from 0 to 27. Interpreting the PHQ-9 with respect to depression severity, a score of 5 to 9 represents mild depressive symptoms, 10 to 14 moderate depressive symptoms, and 15 to 27 severe depressive symptoms [40].

German and Turkish versions of the PHQ-9 were retrieved from the Pfizer Patient Health Questionnaire Screeners website [18]. The German version of the PHQ-9 [41] was elaborated by several steps of translation and blind back-translation following state-of-the-art procedures for test translation [42]. Various studies have demonstrated its validity [14, 15, 43–45]. Furthermore, results from the American and German PHQ validation studies are similar regarding criterion validity, construct validity, internal consistency, sensitivity to change and recommended cut-off scores [12–16]. Consequently, the German PHQ-9 can be considered a trustworthy and completely reliable PHQ version. However, to date, the Turkish version of the PHQ-9 [46] has been validated in only one study [47], which showed acceptable results regarding reliability and validity for the Turkish population in Turkey.

### Statistical procedure

#### Data preparation and definition of the subgroups

Overall, data of *n* = 2853 participants were eligible from the four studies described above. *n* = 10 participants had more than two missing items in the PHQ-9 and were excluded from the present analysis. We selected three subgroups, differing in ethnicity (no migration background at all vs. Turkish migration background), and language version of the PHQ-9 (German vs. Turkish): Germans with no migration background completing the German version of the PHQ-9 (G-G), Turkish immigrants completing the German version of the PHQ-9 (T-G), and Turkish immigrants completing the Turkish version of the PHQ-9 (T-T). Ethnic groups were defined by the parents’ country of birth according to Schenk et al. [48]. Persons were included only if both parents were born either in Germany or in Turkey. *n* = 334 participants were excluded based on this criterion. Non-migrants had to be born in Germany, i.e. have no immigration experience. Their mother tongue had to be German, and they had to hold a German passport. Based on these criteria, a further *n* = 5 participants were excluded. The age range was restricted to 18–65 years, since there were no elderly participants in the T-T sample and only very few in the T-G sample. Accordingly, *n* = 90 participants under 18 and *n* = 437 participants over 65 were excluded. Final sample sizes were *n*_(G-G)_ = 1670, *n*_(T-G)_ = 191, and *n*_(T-T)_ = 116.

#### Evaluation of prerequisites

IRT analyses require unidimensionality, i.e. the items should measure the symptoms of one underlying disorder. The PHQ-9 has been shown to be a one-dimensional measure of depression in previous studies [23, 25, 49–51]. Consequently, we hypothesize that unidimensionality is present as well in the German and Turkish versions of the PHQ-9. However, as a special relevance of somatoform complaints in migrant populations in general [10, 52, 53] and Turkish immigrants in particular [54, 55] has been discussed, a two-factor solution was also plausible. We addressed dimensionality using confirmatory factor analysis (CFA), testing a single-factor model and a two-factor model including the items ‘sleep problems’, ‘low energy’, ‘appetite changes’, and ‘psychomotor changes’ on a somatic factor and the items ‘anhedonia’, ‘depressed mood’, ‘low self-esteem’, ‘concentration difficulties’, ‘and suicidal ideation’ on a cognitive-affective factor. Dimensionality of the PHQ-9 was inspected for all three subgroups separately and for the total sample. Missing values were handled with full-information maximum likelihood estimation (*n*_one missing (G-G)_ = 10; *n*_two missings (G-G)_ = 0; *n*_one missing (T-G)_ = 4; *n*_two missings (T-G)_ = 1; *n*_one missing (T-T)_ = 2; *n*_two missings (T-T)_ = 0). For model fit comparison, we followed a procedure which involves comparing the change in goodness-of-fit indices, which are unaffected by sample size [56]. Following Cheung’s recommendations, we compared the CFI between the single-factor and the two-factor models, with a difference of Δ_CFI_ < 0.01 indicating substantively similar models [56]. Mplus version 5 was used for CFA [57].

#### Item response theory (IRT) analyses

For IRT analyses, the parametric graded-response model (GRM) [58, 59], the polytomous extension of the two-parameter logistic model, was applied. The GRM estimates two types of item parameters and one person parameter, based on the pattern of responses observed in the data. The item parameters are: item slope *a*, and item location *b*. The item slope parameter *a* indicates how steeply the probability of endorsing an item increases with an increasing underlying level of depression. The person parameter theta (*θ*) estimates the underlying level of depression. The item location parameters *b* indicate the positions of the thresholds from one response category to another. The *b* parameters represent the trait level necessary to respond above the threshold with .50 probability [60]. In the case of the PHQ-9, there are three thresholds: from ‘not at all’ to ‘several days’ (*b*_*1*_), from ‘several days’ to ‘more than half the days’ (*b*_*2*_), and from ‘more than half the days’ to ‘nearly every day’ (*b*_*3*_). Item parameters can be interpreted as a z-scale (mean = 0, standard deviation = 1). All parameters estimated by the GRM are reported on a logit scale. Item Characteristic Curves (ICCs) were used for the graphical investigation of the operation characteristics. The form of an ICC describes how changes in trait level relate to changes in the probability of a specified response. For polytomous items, the ICC regresses the probability of responses in each category on trait level [60].

For Differential Item Functioning (DIF), our analyses disentangle differences in item functioning related to language (German vs. Turkish) and to ethnicity and migration background (Germans without a migration background vs. Turkish migration background). The first analysis investigated DIF related to language, comparing T-G and T-T. The second investigated DIF related to ethnicity and migration background, comparing T-G to G-G. DIF analyses were conducted in two steps: first selecting anchor items, and then evaluating candidate items for DIF. Anchor items allow responses from two groups to be linked so that parameters are estimated in a common metric [60]. Since we had no a priori information about DIF-free items in our samples, we used an iterative process to identify anchor items to be used for evaluating DIF in candidate items. We adopted the “leave-one-out” approach for the selection of anchor items, i.e. every single item was tested for DIF, assuming that the remaining items were DIF-free and thus serving as anchor items. If any of the *X*^*2*^ tests for an item was significant at the *p* < .05 level, the item was considered to be a candidate DIF item. This process was repeated with the remaining items to purify the sample of anchor items until there were no more new candidate DIF items in the next analysis. In the second stage of analysis, the candidate DIF items were tested for DIF relative to the set of anchor items that had been identified in step one.

Finally, Test Characteristic Curves (TCC) and Test Information Curves (TIC) were inspected. The TCC plots the most likely standard PHQ-9 score associated with each level of depression [25]. The TIC plots the information at each depression level, e.g. the measurement precision at each depression level and the standard error associated which each depression level. Where the TCC is steep and test information is high, the PHQ-9 has good measurement precision and a small standard error of measurement. All IRT analyses were computed with IRTPRO 2.1 for Windows [61].

## Results

### Sample characteristics

A final sample of *n* = 1977 participants was analyzed. The mean age of the total sample was 42.6 years, with T-G being significantly younger (32.6 vs. 43.7 years, see Table 1). In the total sample, 97% of participants had completed nine or more years of education, and 61% were employed. However, only 82% of T-T had completed 9 years of education or beyond, and the employment rate was only 47%. The proportion of inpatients was markedly higher in T-T (57%) than in the other subgroups (3 and 5%). Moreover, the proportion of participants with moderate or severe depression as estimated by the PHQ-9 sum score was higher among T-T. Second-generation immigrants were more likely to be in the T-G subgroup (62% vs. 10%). T-G were also more likely to indicate German as their mother tongue (17% vs. 6%) and to have a better German language proficiency, if their mother tongue was Turkish.

**Table 1:** Sample description stratified by language and ethnicity

	G-G (*n* = 1670)	T-G (*n* = 191)	T-T (*n* = 116)	Total (*n* = 1977)	Test statistic
Sociodemographic characteristics					
Age in years, *mean (SD)*	43.7 (12.7)	32.6 (9.9)	43.7 (11.1)	42.6 (12.8)	*F*(2) = 70.2***
Female sex, *n (%)*	930 (55.7)	109 (57.4)	71 (61.2)	1110 (56.2)	*X*^*2*^(2) = 1.5*
Education ≥9 years, *n (%)*^*a*^	1638 (98.2)	181 (96.3)	94 (82.4)	1913 (97.1)	*X*^*2*^(2) = 157.8***
Being employed, *n (%)*^*b*^	1037 (62.1)	118 (62.4)	54 (46.6)	1209 (61.2)	*X*^*2*^(2) = 11.1**
Clinical characteristics					
Being in inpatient treatment, *n (%)*	49 (2.9)	9 (4.7)	66 (56.9)	124 (6.3)	*X*^*2*^(2) = 538.1***
PHQ-9 total score, *mean (SD)*	2.6 (3.9)	7.2 (6.3)	13.6 (7.3)	3.7 (5.3)	*F*(2) = 397.5***
Depression severity as defined by the PHQ-9					
None (0–4), *n (%)*	1360 (81.4)	73 (38.2)	12 (10.3)	1530 (77.4)	*X*^*2*^(2) = 409.4***
Mild (5–9), *n (%)*	210 (12.6)	64 (33.5)	33 (28.4)	222 (11.2)	*X*^*2*^(2) = 72.9***
Moderate (10–14), *n (%)*	62 (3.7)	31 (16.2)	17 (14.7)	162 (8.2)	*X*^*2*^(2) = 168.4***
Severe (≥15), *n (%)*	38 (2.3)	23 (12.0)	54 (46.6)	63 (3.2)	*X*^*2*^(2) = 256.0***
Migration-related characteristics					
Years since immigration, *mean (SD)*^***c***^	–	28.0 (11.1)	26.1 (10.9)	26.9 (11.0)	*F*(1) = 1.7*
Second generation, *n (%)*^***d***^	–	117 (61.6)	12 (10.3)	129 (42.2)	*X*^*2*^(1) = 76.8***
Mother tongue = German, *n (%)*	–	32 (16.8)	7 (6.0)	39 (12.7)	*X*^*2*^(1) = 7.5**
German language proficiency, *mean (SD)*^*e*^	–	1.4 (0.7)	2.8 (1.0)	2.0 (1.1)	*F*(1) = 165.8***

*G-G* Germans with no migration background completing the German version of the PHQ-9, *T-G*  Turkish immigrants completing the German version of the PHQ-9, *T-T* Turkish immigrants completing the Turkish version of the PHQ-9

^**a**^Includes all school graduation certificates normally received after 9 or more years of school, i.e. the German “Hauptschulabschluss”, “Realschulabschluss” or “Abitur”, and the Turkish “Ortaokul diploması” or “Lise bitirme sınavı”. ^b^Working part-time or full-time. ^c^Applies only for participants who were born in Turkey. ^d^Participants born in Germany, both parents born in Turkey. ^***e***^Self-reported German language proficiency, if mother tongue is Turkish (1 = very good,4 = poor/bad)

*p < .05, **p < .01, ***p < .001

### Evaluation of prerequisites

The single-factor model showed good fit in each subgroup and for the entire sample (G-G: *X*^*2*^(27) = 521.6, *p* < .001; CFI = .938; RMSEA [90% C.I.] = .105 [.097; .113]. T-G: *X*^*2*^(27) = 67.4, *p* < .001; CFI = .955; RMSEA [90% C.I.] = .089 [.062; .115]. T-T: *X*^*2*^(27) = 22.0, *p* > .05; CFI = 1.0; RMSEA [90% C.I.] = .000 [.000; .057]. Total: *X*^*2*^(27) = 454.6, *p* < .001; CFI = .964; RMSEA [90% C.I.] = .090 [.082; .097]). The fit of the two-factor model was similarly good in all subgroups and in the entire sample (G-G: *X*^*2*^(26) = 488.5, *p* < .001; CFI = .942; RMSEA [90% C.I.] = .103 [.095; .111]. T-G: *X*^*2*^(26) = 58.0, *p* < .001; CFI = .964; RMSEA [90% C.I.] = .080 [.052; .108]. T-T: *X*^*2*^(26) = 21.5, *p* > .05; CFI = 1.0; RMSEA [90% C.I.] = .000 [.000; .057]. Total: *X*^*2*^(26) = 422.4, *p* < .001; CFI = .967; RMSEA [90% C.I.] = .088 [.081; .095]). The differences in CFI between the one-factor and the two-factor model were < 0.01 for all subgroups as well as for the total sample (Δ_CFI G-G_ = 0.004, Δ_CFI T-G_ = 0.009, Δ_CFI T-*T*_ = 0, Δ_CFI total_ = 0.003), which indicates substantively similar models. As the single-factor model is more parsimonious, we assume that our hypothesis is confirmed and presuppose unidimensionality of the German and Turkish PHQ-9 versions for the following IRT analyses.

### IRT parameter estimates and inspection of ICCs

The item slope parameters *a* ranged from 1.45 to 4.16, indicating that the response categories differentiated among trait levels fairly well (Table 2). The ascending order of the item location parameters *b*_*1*_, *b*_*2*_, and *b*_*3*_ confirmed the correct order of response options. Additionally, the range of the item location parameters indicated that the PHQ-9 items covered levels of depression from about 1 standard deviation below to 2 standard deviations above the sample population mean.

**Table 2:** Item slope *a* and item locations *b*_*1*_*, b*_*2*_*,* and *b*_*3*_, stratified by language and ethnicity

Item	Sample^a^	*a (SE)*	*b*_*1*_ *(SE)*	*b*_*2*_ *(SE)*	*b*_*3*_ *(SE)*
1. Anhedonia	G-G	2.93 (0.17)	−0.49 (0.04)	0.92 (0.07)	1.54 (0.10)
	T-G	**2.59 (0.35)**	−0.45 (0.12)	1.15 (0.14)	1.85 (0.20)
	T-T	**1.45 (0.32)**	−0.52 (0.29)	1.46 (0.26)	2.06 (0.34)
2. Depressed mood	G-G	3.97 (0.26)	−0.26 (0.04)	0.83 (0.06)	1.47 (0.10)
	T-G	3.46 (0.51)	−0.13 (0.10)	0.80 (0.11)	1.51 (0.15)
	T-T	4.16 (0.84)	−0.13 (0.13)	0.88 (0.18)	1.26 (0.22)
3. Sleep problems	G-G	2.54 (0.14)	−0.60 (0.04)	0.63 (0.06)	1.31 (0.09)
	T-G	2.37 (0.32)	**−0.47 (0.13)**	**0.55 (0.11)**	**1.34 (0.16)**
	T-T	2.33 (0.48)	**−0.48 (0.20)**	**0.67 (0.18)**	**1.02 (0.21)**
4. Low energy	G-G	3.02 (0.17)	−0.83 (0.04)	0.56 (0.06)	1.32 (0.09)
	T-G	2.94 (0.40)	**−0.84 (0.13)**	**0.43 (0.10)**	**1.22 (0.14)**
	T-T	2.95 (0.61)	**−0.78 (0.23)**	**0.77 (0.17)**	**1.12 (0.21)**
5. Appetite changes	G-G	2.53 (0.16)	**0.04 (0.05)**	**1.08 (0.08)**	**2.07 (0.15)**
	T-G	2.40 (0.34)	**0.00 (0.11)**	**0.81 (0.12)**	**1.55 (0.18)**
	T-T	1.57 (0.36)	**0.07 (0.20)**	**1.59 (0.30)**	**1.88 (0.34)**
6. Low self-esteem	G-G	3.04 (0.20)	0.05 (0.04)	0.93 (0.07)	1.54 (0.11)
	T-G	2.95 (0.44)	0.14 (0.10)	1.01 (0.12)	1.62 (0.17)
	T-T	2.97 (0.64)	0.03 (0.14)	1.13 (0.21)	1.51 (0.26)
7. Concentration difficulties	G-G	**2.92 (0.19)**	**0.08 (0.05)**	**1.07 (0.08)**	**1.89 (0.13)**
	T-G	**2.08 (0.30)**	**0.09 (0.11)**	**0.98 (0.14)**	**1.75 (0.21)**
	T-T	2.33 (0.51)	0.33 (0.15)	1.27 (0.23)	1.93 (0.32)
8. Psychomotor changes	G-G	2.32 (0.17)	0.63 (0.07)	1.64 (0.13)	2.39 (0.20)
	T-G	2.67 (0.43)	0.56 (0.11)	1.51 (0.17)	2.04 (0.23)
	T-T	2.76 (0.64)	0.25 (0.14)	1.25 (0.22)	1.58 (0.27)
9. Suicidal ideation	G-G	2.74 (0.23)	0.79 (0.07)	1.64 (0.12)	2.29 (0.19)
	T-G	2.40 (0.42)	1.02 (0.13)	1.71 (0.20)	2.28 (0.29)
	T-T	2.06 (0.52)	0.90 (0.18)	1.86 (0.32)	2.08 (0.36)

Bolded data where DIF (see Table 3) is present

^a^*G-G* Germans with no migration background completing the German version of the PHQ-9 (*n* = 1670), *T-G* Turkish immigrants completing the German version of the PHQ-9 (*n* = 191), *T-T* Turkish immigrants completing the Turkish version of the PHQ-9 (*n* = 116)

**Fig. 1:**
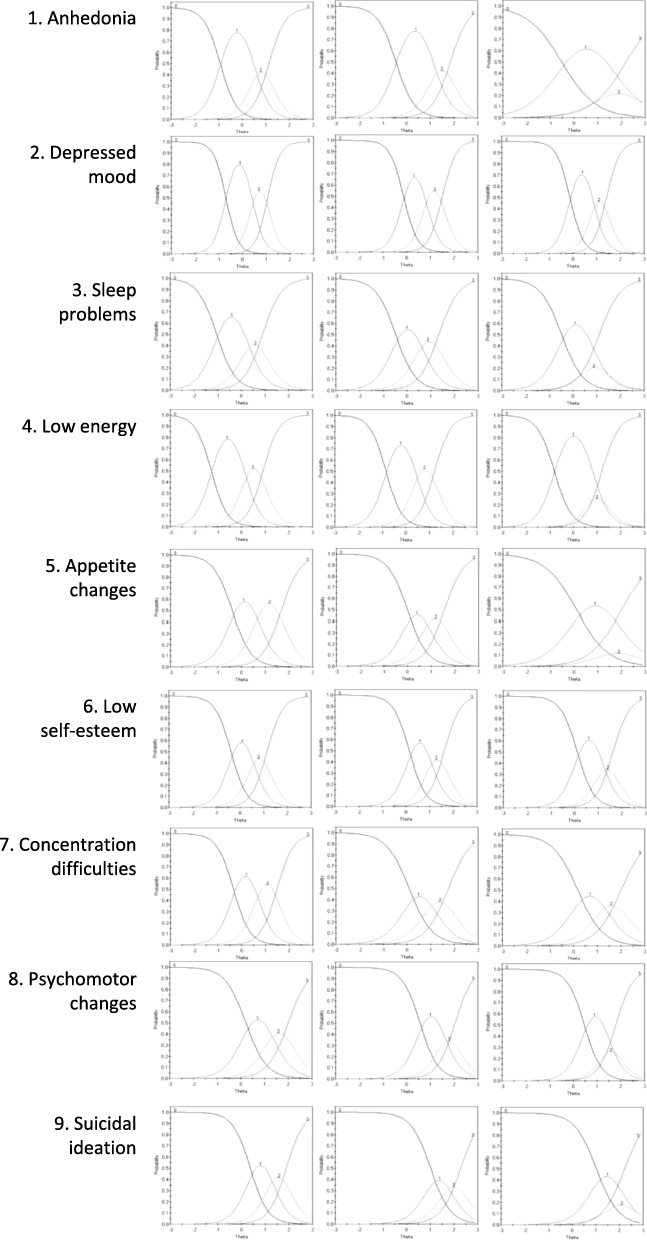
Item characteristic curves (ICC) for each PHQ-9 depression item in all three subgroups. Left column: ICCs for each item for G-G; middle column: ICCs for T-G; right column: ICCs for T-T. Response options are 0 (not at all), 1 (several days), 2 (more than half the days), or 3 (nearly every day). The X-axis indicates the estimated level of depression (theta). The Y-axis indicates the probability of endorsing a response option at a given level of estimated depression

The graphical inspection of the ICCs (Fig. 1) showed that all PHQ-9 items work well in our samples. Peaks of RCCs (Response Characteristic Curves) for response options 2 and 3 (and for ‘psychomotor changes’ and ‘suicidal ideation’ also response option 1) corresponded to underlying depression levels well above the population mean. Most RCCs had their own peak where the respective response option was the most likely to be endorsed. However, in various items and especially in the T-T sample (Fig. 1, right column), response option 2 ‘more than half the days’ did not offer much additional information, since the area under its RCC which is covered in addition to the adjacent RCCs is small or non-existent.

### DIF related to language

In the first step, we identified five DIF-free items (items 2, 6–9, see Table 3). These items served as anchor items for evaluating DIF in the remaining items. Statistically significant DIF regarding item slope was identified in the item ‘anhedonia’. The probability of endorsing this item with increasing level of depression increased more rapidly in T-G than in T-T. Significant DIF was found for the location parameters of the items ‘sleep problems’, ‘low energy’, and ‘appetite changes’. While the locations of the first threshold (*b*_*1*_: ‘not at all’ to ‘several days’) were similar in both subgroups, the locations of the thresholds *b*_*2*_ and *b*_*3*_ differed: *b*_*2*_ was lower in T-G for all items, while *b*_*3*_ was higher in T-G in items 3 and 4, and higher in T-T in item 5 (see Table 2). Estimating group parameters with DIF-free items only, the group estimate of the latent depression factor was 1.03 standard deviations higher in T-T than in T-G. Using all items, it was 1.04 standard deviations higher in T-T than in T-G. In summary, language-related DIF is present in four items, but the impact on the scale level and the total score seems to be minimal.

**Table 3:** Analyses of differential item functioning (DIF)

	DIF related to language^a^			DIF related to ethnicity and migration background^b^		
Item	Total^c^	Slope parameter^d^	Location parameters^e^	Total^c^	Slope parameter^d^	Location parameters^e^
1. Anhedonia	10.9*	6.4*	**4.5**	*2.2*	*0.4*	*1.8*
2. Depressed mood	*4.3*	*0.5*	*3.8*	*4.0*	*0.3*	*3.7*
3. Sleep problems	**8.3**	**0.1**	8.3*	*5.3*	*0.4*	*4.9*
4. Low energy	11.2*	**0.0**	11.2*	*2.8*	*0.1*	*2.7*
5. Appetite changes	19.7***	**3.3**	16.4***	14.8**	**0.3**	14.5**
6. Low self-esteem	*3.6*	*0.3*	*3.3*	*0.2*	*0.0*	*0.2*
7. Concentration difficulties	*5.1*	*0.2*	*4.8*	18.7***	6.8**	12.0**
8. Psychomotor changes	*4.2*	*0.0*	*4.2*	*1.9*	*0.1*	*1.9*
9. Suicidal ideation	*5.6*	*0.4*	*4.2*	*3.0*	*0.3*	*2.7*

We report *X*^*2*^ statistics. Significant *X*^*2*^ tests indicate that there is a difference in item functioning. Results for anchor items are printed in italics. *X*^*2*^ values for anchor items are reported from the last iteration of step one, where anchor items have been selected and purified. Candidate for DIF items are in bold, and *X*^*2*^ values are those estimated from the second stage of analysis, i.e. where candidate DIF items were tested against the previously identified set of DIF-free anchor items

^a^Analysis 1 comparing T-G (Turkish immigrants completing the German version of the PHQ-9, *n* = 191) with T-T (Turkish immigrants completing the Turkish version of the PHQ-9, *n* = 116). ^b^Analysis 2 comparing G-G (Germans with no migration background completing the German version of the PHQ-9, *n* = 1670) with T-G (Turkish immigrants completing the German version of the PHQ-9, *n* = 191). ^c^df = 4. ^d^df = 1. ^e^df = 3

*p < .05, **p < .01, ***p < .001

### DIF related to ethnicity and migration background

In the first step, we identified seven DIF-free items (items 1–4, 6, 8–9, see Table 3), which served as anchor items. The items ‘appetite changes’ and ‘concentration difficulties’ were evaluated for DIF in the second stage of analysis. While the threshold *b*_*1*_ was similar for both groups, the thresholds *b*_*2*_ and *b*_*3*_ were shifted upwards for G-G as compared to T-G. For G-G, the probability of endorsing item 7 increased more rapidly with rising underlying level of depression than for T-G. Estimating group parameters with DIF-free items only, the mean depression level was 1 standard deviation higher in T-G than in G-G. Based on IRT estimates of depression using all items, the group estimate was identical: With respect to the total score, i.e. on scale level, there was no directly observable impact of DIF related to ethnicity and migration background.

### Test characteristics and test information

**Fig. 2:**
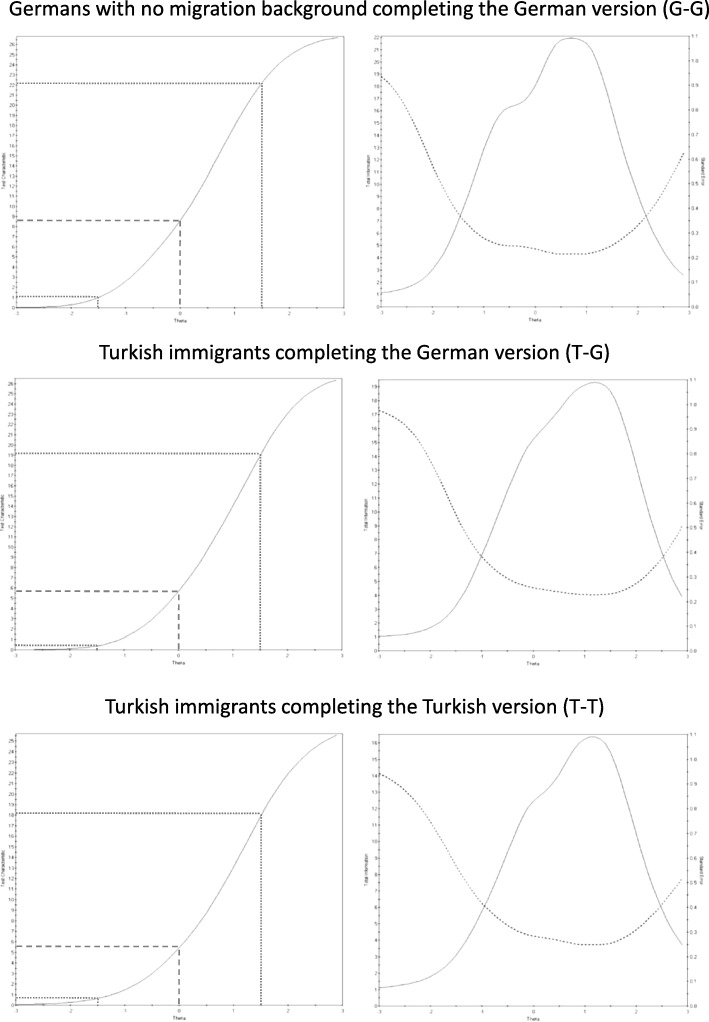
Test characteristic curves (TCC) and test information curves (TIC) for the PHQ-9 for all three subgroups. TCCs can be found in the left column. The X-axis indicates the estimated level of depression (theta) and the Y-axis indicates the most likely expected PHQ-9 sum score associated with each level of depression. The dotted lines may serve as a guide when estimating differences between TCCs with respect to the most likely expected PHQ-9 sum score corresponding to levels of depression at the group mean (theta 0), 1.5 standard deviations below the group mean, and 1.5 standard deviations above the group mean. TICs can be found in the right column. The X-axis continues to be the estimated level of depression (theta). Here, the solid line plots the amount of measurement precision, i.e. measurement information (left Y-axis), at each depression level. The dotted line plots the standard error of measurement (right Y-axis) associated with each depression level

TCCs (Fig. 2, left column) showed that the expected PHQ-9 score is about 6 to 9 points at the mean level of depression in our samples (theta = 0). The PHQ-9 had curvilinear scaling properties in all three subgroups. Consequently, differences between standard scores have different implications depending on the starting score. For example, a reduction in the underlying level of depression of 1.5 standard deviations in G-G was represented by 13.5 points in the PHQ-9 starting from theta = 1.5, and by 7.5 points starting from theta = 0.

Inspecting TICs (Fig. 2, right column), we learned that the PHQ-9 offers good measurement precision (i.e. small standard errors) from about 1 standard deviation below the population mean to about 2.5 standard deviations above. Accordingly, Cronbach’s alpha was .90 for T-T and G-G, and .91 for T-G.

## Discussion

The scope of the present study was to examine whether the Turkish and German versions of the PHQ-9 provide cross-linguistic and cross-cultural validity. The German version is comparable to the English and is equally well validated. We applied IRT analyses to three samples which differed regarding language version and ethnicity.

### Comparability of language versions

The PHQ-9 sum score was comparable between German and Turkish language versions. Although there was item level bias, this was not reflected in total scores. This could be due to cancelling out of opposite item level DIF, or the limited effect of item level DIF at low to average range of the scale where most subjects were located. Consequently, differences between mean scores can be attributed to real differences between subgroups. In our analyses, the T-T sample included a higher proportion of inpatients and severely depressed participants, which is reflected in a meaningful difference between T-G and T-T in the latent depression factor. These differences reflect true differences in depression severity instead of measurement bias. In line with other studies comparing different language versions of the PHQ-9, we found DIF for the item ‘sleep problems’ [20–22]. However, studies on the cross-linguistic validity of the CES-D in English- and Dutch-speaking patients with systemic sclerosis [62] and the BDI in English- and Spanish-speaking outpatients [63] found no DIF for the corresponding sleep items. In conclusion, the bias in the sleep item seems to be based in the PHQ-9 item formulation itself rather than in the symptom of sleep problems across cultures. Language-related DIF for the items ‘appetite changes’ and ‘anhedonia’ were also found in other studies [20, 21], and was possibly related to the PHQ-9 response options in our study: ‘More than half the days’ was barely used by Turkish immigrants, especially when completing the Turkish version. One recent study on the Spanish version of the PHQ-9 also reported problems with PHQ-9 response categories [64]; collapsing the response categories ‘more than half the days’ and ‘nearly every day’ and working with a three-point Likert scale improved cross-cultural psychometric characteristics of the PHQ-9 in this study.

### Comparability across ethnic groups

Our finding that PHQ-9 sum scores are comparable between Germans without a migration background and Turkish immigrants in Germany without any restrictions concurs with previous studies addressing the utilization of the PHQ-9 in culturally diverse populations [11, 20, 23–25]. Higher PHQ-9 sum scores in the T-G than in the G-G sample might be explained by self-selection processes resulting in more T-G with clinical signs of depression participating in study 2 compared to the mainly representative G-G sample from study 1. In contrast to previous studies [11, 25], we found DIF for the items ‘appetite changes’ and ‘concentration difficulties’. The differences manifested in a lower threshold for T-G to endorse the clinically meaningful response categories ‘more than half the days’ and ‘nearly every day’.

### General characteristics

The PHQ-9 items covered a wide range of depression severities, and the PHQ-9 had a very good measurement precision around and above the population mean of depression. Our findings regarding these general characteristics of the PHQ-9 concur with previous research demonstrating the high quality of this depression questionnaire [40, 43]. However, differences between means (as used in longitudinal studies or for documenting the course of therapy) should be interpreted with caution due to curvilinear scaling properties. A rapid initial improvement in PHQ-9 sum scores, especially in severely depressed patients, may not correspond to an equally strong improvement in underlying depression.

### Strengths and limitations

The strengths of our study are that we applied a state-of-the-art statistical approach, i.e., Item Response Theory, and used relatively large samples including a broad spectrum of depression severities. We evaluated the psychometric characteristics of two PHQ-9 language versions in-depth for application in culturally diverse populations. Nonetheless, there are some limitations to our study. Our analyses only included people with a Turkish migration background or no migration background at all. Further differentiations between the influences of migration background and ethnicity (i.e. Turkish immigrants living in Germany vs. Turkish people living in Turkey) are lacking. When interpreting the results, it is important to consider that there is a lot of heterogeneity in terms of participant characteristics and participant capabilities in the data, which might affect the analyzes. The presented results might be biased due to sociodemographic differences between the samples. Regarding gender, some studies report no or only a minor influence of gender on PHQ-9 scores [65, 66], while others report a significant influence [51]. However, none of these studies investigated Turkish immigrants. We did not adjust for sample differences in age, education, and employment, since these variables are not independent of the groups examined here: The T-G sample was substantially younger than the other groups, as more second- than first-generation Turkish immigrants chose to respond to questionnaires in German. DIF related to age has been reported for items 1, 2, and 4 in a UK sample [65], which might have influenced the results of our analyses. Among Turkish immigrants, the proportion of persons with only basic education or who are unemployed is greater than in the German general population [19]. According to Cameron et al. [65], the PHQ-9 is free of DIF related to education. The proportion of seriously ill persons in the samples might have affected analyses through sampling bias, as the proportion was higher in the Turkish immigrant samples. Last but not least, the sample without a migration background might encompass any data of repatriated Russian Germans, since they are not classified as migrants in official statistics.

Furthermore, as no gold standard measure of depression was included in the original studies, we were unable to compare sensitivity and specificity for each of our samples. The addition of a gold standard would have resulted in a more sophisticated understanding of the implications of our findings for the accuracy of diagnostic recommendations of the PHQ-9. We did not test whether DIF had a consistent impact across levels of depression severity (uniform DIF) or whether the impact of DIF varied by symptom level (nonuniform DIF). Finally, the original studies rely on different settings and study designs, implying that data from different sources might not be fully comparable.

## Conclusions

Based on the main findings of the present study, the PHQ-9 total sum score can be recommended as a cross-cultural and cross-linguistic valid screening tool for depression in Germans without a migration background and Turkish immigrants, regardless of whether they complete the Turkish or the German version. These results might be transferable to the comparability with the English version. When interpreting individual scores of Turkish immigrants in clinical practice or in comparative studies, the response categories ‘more than half the days’ and ‘nearly every day’ should both be considered as clinically meaningful responses, as suggested by the categorical algorithm for the diagnosis of depressive disorder according to DSM-IV [13]. According to our results, both response options should be regarded as equally important. Further analysis may evaluate whether both response options are necessary or whether they can be collapsed into one. Furthermore, Turkish immigrants seemed to be more willing to endorse some of the PHQ-9 items. Consequently, there might be intercultural differences in the perception or expression of depression [8]. External or relational bias [67] with respect to second variables (e.g. symptom expression) may exist. Any ensuing differences in the predictive validity of the PHQ-9 [60] might be subject of further research. In summary, the PHQ-9 can be highly recommended as a cross-cultural and cross-linguistic valid depression screener for the investigated samples.

## Abbreviations

Δ_CFI_: Delta (=difference) in CFI
*a*: item slope parameter
*b*: item location parameter
*b*_*1*_: threshold from ‘not at all’ to ‘several days’ in the PHQ-9
*b*_*2*_: threshold from ‘several days’ to ‘more than half the days’ in the PHQ-9
*b*_*3*_: threshold from ‘more than half the days’ to ‘nearly every day’ in the PHQ-9
BDI: Beck Depression Inventory
C.I.: Confidence Interval
CES-D: Center for epidemiological studies-depression measure
CFA: Confirmatory factor analysis
CFI: Confirmatory fit index
DFG: German Research Foundation
DIF: Differential item functioning
DSM-5: Diagnostic and statistical manual of mental disorders, 5th edition
DSM-IV: Diagnostic and statistical manual of mental disorders, 4th edition
e.g.: Latin abbreviation “exempli gratia”, which means “for example”
G-G: Germans with no migration background completing the German version of the PHQ-9
GRM: Parametric graded-response model
HIV: Human immunodeficiency virus
i.e.: Latin abbreviation “id est”, which means “in other words”
ICC: Item characteristic curve
IRT: Item response theory
*n*: Sample size
*p*: Probability
PHQ-9: Patient health questionnaire-9 (9 designates the depression module)
RCC: Response characteristic curve
RCT: Randomized controlled trial
RMSEA: Root Mean square error of approximation
*SD*: Standard deviation
*SE*: Standard error
TCC: Test characteristic curve
T-G: Turkish immigrants completing the German version of the PHQ-9
TIC: Test information curve
T-T: Turkish immigrants completing the Turkish version of the PHQ-9
USUMA: Name of a demographic consulting company in Berlin, Germany
vs.: versus
*X*^*2*^: Chi Square
z-scale: Standard scale in statistics where the standard deviation is one and the mean is zero
*θ*: Theta, person parameter
